# A longterm prospective follow-up study of resurfacing miniprosthesis suitable for patients above sixtyfive years with localized cartilage lesions or early osteoarthritis in the knee

**DOI:** 10.1186/s40634-020-00308-9

**Published:** 2020-12-06

**Authors:** Jens Ole Laursen, Martin Lind, Christian Backer Mogensen, Helene Skjøt-Arkil

**Affiliations:** 1 Department of Emergency Medicine, Hospital of Southern Jutland, Vimmelskaftet 16, 6470 Sydals, Denmark; 2 Department of Orthopedic Surgery, Hospital of Southern Jutland, Sydals, Denmark; 3 Institute of Regional Health Research, University of Southern Denmark, Odense, Denmark; 4 Department of Orthopedic Surgery, Aarhus University Hospital, Aarhus, Denmark

**Keywords:** Condylar implant, Femoral resurfacing, Cartilage injury, Large cartilage lesions, Early osteoarthritis, Small implants, Knee prosthesis

## Abstract

**Purpose:**

The aim of the study was to investigate the long-term outcomes of the Focal Femoral Condyle Resurfacing Prosthesis for treatment of localized cartilage lesion in patients > 65 years.

**Methods:**

This was a prospective case series study. Non-reopererated patients initially treated with resurfacing condylar miniprothesis (HemiCAP/UniCAP) were evaluated clinically and radiographically at 7–10 years follow-up (mean 9 years). The clinical examination included the Knee Society Score (KSS) and Visual Analogue Scale (VAS) pain score and EQ5D. The radiographic examination included the Kellgren-Lawrence (KL) grade for investigate of OA progression. A comparison analysis of the preoperative and follow-up subjective outcome data and a Kaplan-Meier implant survival analysis were performed.

**Results:**

Twenty-three patients were included in the study (9 HemiCAP and 14 UniCAP). There were seven revisions (one HemiCap and six UniCap respectively) (30%) and three patients had died. Follow-up examinations were performed on 10 patients. When comparing follow-up with the preoperative state, there were significant increases in the KSS objective (50.0 ± 8.3) vs. 90.0 ± 6.3)) and KSS function (45.0 ± 11.7) vs. 85.0 ± 4.7)) scores, a decrease in the pain VAS score (7.0 ± 0.9) vs. (4.0 ± 1.9)). Radiographic evaluation demonstrated increase in osteoarthritis development with a KL medial score (2.0 ± 0.6) and KL lateral score (1.4 ± 0.6) vs. (2.0 ± 0.9)).The EQ5D-score was 86 ± 8.4 and patients Health-score was 85 ± 18).

**Conclusions:**

Resurfacing implant treatment for early OA in patients above 65 years can require revision to knee arthroplasty in 30% of patients. But in patients that are not revised long-term improvements in subjective clinical outcome was demonstrated. This suggests that even elderly patients with isolated cartilage lesions or early OA might benefit from the limited invasive resurfacing implant treatment.

**Level of evidence:**

IV

## Introduction

Middle aged to elderly patients with knee pain and disability caused by localized cartilage lesions or early osteoarthritis (OA) can be challenging to treat, when radiographic and clinical status does indicate treatment with a unicompartmental knee arthroplasty (UKA) or total knee arthroplasty (TKA). Thus, these patients may pursue nonoperative treatment modalities, such as physiotherapy, weight loss, analgesics and activity modifications [19]. In order to fill an existing treatment gap in the middle-aged patient, small condylar implants were developed for treatment of symptomatic cartilage lesions that leave the unaffected joint compartments alone. The first were onlay miniprostheses [6, 11], followed by the later development of inlay miniprostheses, which not only addressed the size of the cartilage lesion but also the patient-specific curvatures of the knee [2–6, 8, 10–12, 15, 17, 18]. In 2003, an anatomic metallic implant for femoral resurfacing called the Focal Femoral Condyle Resurfacing Prosthesis (HemiCAP) was introduced for full-thickness smaller condylar lesions (both femoral and trochlear). It was first approved for use in Denmark in 2006, with a 2015 publication describing its specific indications [17]. Only limited evidence of the clinical outcomes and failure rates has been presented for the HemiCAP. Two case series of approximately 20 patients [5, 11] with varying osteochondral pathologies demonstrated reduced pain and improved knee function. Additionally, a study performed in 2015 [15] demonstrated the good clinical outcomes, but with a concerning 23% revision rate based on the medium-term follow-up results (6 years). Recent long-term studies of the UniCAP [17] and HemiCAP [18] found revision-rates at 60% and 40% respectively. The results in these studies were based on patiens aged in their fifties. So far no studies have investigated more long-term outcomes in more elderly patients with moderate sized cartilage lesions treated with the mini-prothesis concept.

The aim of this study was therefore to investigate whether treatment of localized cartilage leasions with Focal Femoral Condyle Resurfacing Prosthesis in elderly patients above 65 years can lead to relevant improvement in clinical outcome. It was hypothesized that treatment would reduce pain and improve knee function in long-term treatment even in elderly patients.

## Material and methods

### Study design and setting

This was a prospective case-series study of patients treated with femoral resurfacing between 2007 and 2013 [15, 16]. It was reported according to principles outlined in the Strengthening the Reporting of Observational Studies in Epidemiology statement [20].

### Participants

From 2007 to 2013, 25 operations were done in the group of elder patients with minor cartilage lesions or incipient OA. Two patient records were lost. The inclusion criteria were treatment symptomatic cartilage lesions at the femoral condyle or trochlea as demonstrated using magnetic resonance imaging or arthroscopy, with an International Cartilage & Joint Preservtion Society (ICRS) grade of 3–4 and a lesion size of less than 400 mm^2^ for the HemiCAP and exceeding 400 mm^2^ for the UniCAP. There were 6 males and 17 females, with a median age of 72 (66–84) years old. Exclusion criteria were: valgus or varus malalignment > 5 degrees, ligament instability, more than 50% meniscus removal or a body mass index of more than 40. The 23 patients included in this study were followed for mean 9 years.

### Device description

The HemiCAP and UniCAP resurfacing implants are well-described in previously published papers [15, 16].

### Outcome evaluation

Patients that had undergone revision to UKA/THA were identified in the Danish Knee Registry [7]. Those patients, who were not revised, were invited to participate in this study and clinically examined by a senior surgeon if written consent was obtained. OA development were radiographically evaluated by assessing the Kellgren-Lawrence (KL) grade for the medial, lateral and patellofemoral compartments [14]. Subjective outcome were evaluated by the Knee Society Score (KSS) objective and function subscales [13]. Pain was evaluated using a numerical rank scale (0–10), with 10 being the worst possible pain and quality of life subjective evaluation was performed using the EQ5D health score [9].

### Statistical analysis

The demographics and baseline (preoperative) characteristics of the patients were presented as median and interquartile range (IQR) values. Sine most data not were normally distributed a Wilcoxon signed-rank test was used for the paired data comparisons. The Kaplan-Meier survival analysis was used with dropouts (revision or death) as the endpoints and a 95% confidence interval (CI). *P* values of less than 0.05 were considered to be statistically significant. For the statistical analysis, Stata: Data Analysis and Statistical Software for Professionals version 15.1 (StataCorp LLC, College Station, TX, USA) was used. All of the data collected was stored in accordance with the Danish Data Protection Agency requirements. This study was approved by the regional data committee of the Region of South Jutland (# 2008-58-0035).

### Ethical consideration

Written consent to participate in the study was obtained. According to Danish law approval by ethical committee was not necessary for follow-up studies.

## Results

**Fig. 1:**
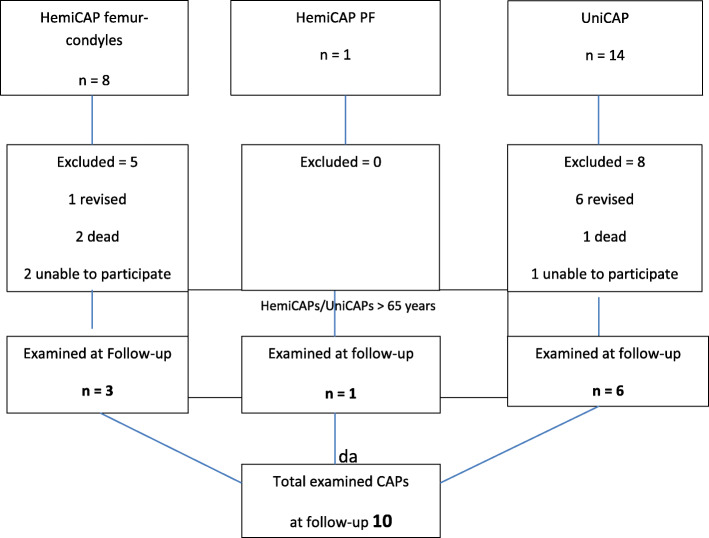
Flowcart of up to 11 years follow-up of HemiCAPs and UniCAPs in 23 patients > 65 years old

Of the 23 CAP procedures, 13 (57%) were excluded from the follow-up due to revisions or death. Four patients were unable to participate (Fig. 1). The mean follow-up time was 9.6 ± 1.4 years) with a range from 7.1 to 11.9 years.

**Fig. 2:**
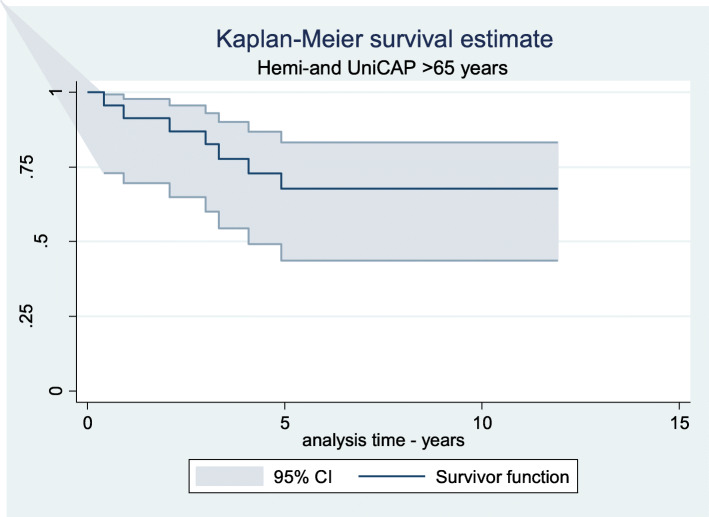
KSS objective- and function subscales in 10 patients at follow-up

**Fig. 3:**
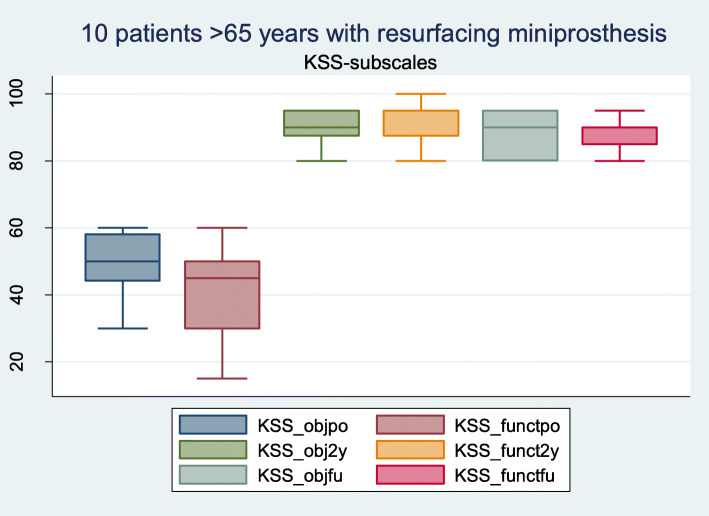
VAS-score in 10 patients at follow-up

The objective and subjective outcomes (KSS) and radiographic and OA evaluations (KL-OA) are shown in Table 1 and Fig. 2. Both the KSS objective and function scores improved significantly from the preoperative scores to the follow-ups at 7–11 years. The pain score was reduced significantly (Fig. 3). The EQ5D at follow-up was median at 86 ± 8.4 and health-score median at 84.5 ± 17.8).

**Table 1:** Up to 11 years follow-up on HemiCAP/ UniCAPs in elder patients > 65 years, with high significant improvements in pain- and function-scores

	Pre-op			follow-up			Comparison
	n	Median	Range	n	Median	Range	*p*-value*
BMI	23	28	+/− 3.7	10	29	+/− 3.8	ns
KSS							
- Objective	23	50	+/− 8.3	10	90	+/− 6.3	*p* < 0.01
- Function	23	45	+/−11.7	10	85	+/− 4.7	*p* < 0.01
Pain score	23	7	+/− 0.9	10	4	+/−1.9	*P* < 0.01
KL score							
- Medial	23	2.0	+/− 0.6	10	2.5	+/− 0.5	*p* < 0.01
- Lateral	23	1.4	+/− 0.6	10	2.0	+/− 0.9	*p* < 0.01

*KSS* Knee Society Scores, *BMI* Body mass index, *KL* Kellgren-Lawence, *ns* Not significant

* Wilcoxon signed-rank test

KL grade increased significantly from the preoperative to follow-up in both chambers (Table 1). Medial chamber KL changed from 2.0 to 2.5 and lateral chamber KL score changed from 1.4 to 2.0.

**Fig. 4:**
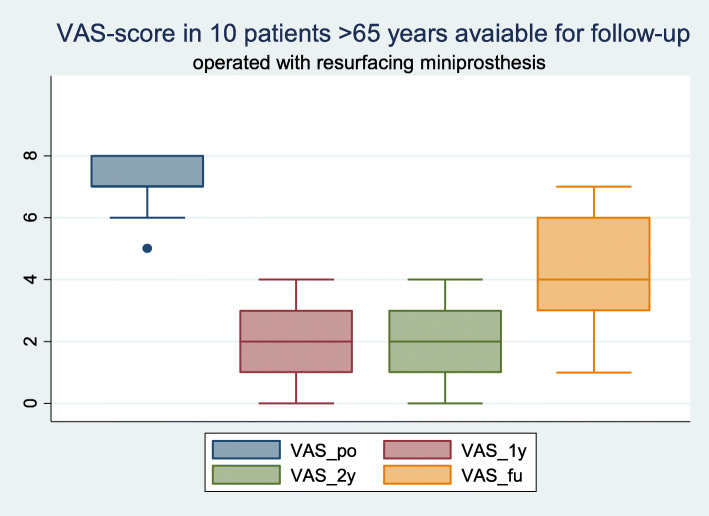
23 patients > 65 years old operated with HemiCAP (9) or UniCAP (14)

Of the 23 CAP procedures, 7 (30%) were revised. Kaplan-Meier survival at 2 years 85% and 5 years 73% and 9 years 70%. No revisions were seen if the prosthesis survived the first 5 years (Fig. 4). The revision causes included increasing pain, disability and OA progression. There were no deep infections or aseptic loosening.

## Discussion

The most primary findings of the present study was the relative high revision rate of 30% during the first 5 years after the mini- prosthesis implantation. Similar results are published previously [15–18], and were consistent with the findings from the Australian and Danish Knee Arthroplasty Registries [1, 7]. An interesting finding of the present study were that no revisions at a late stage from 5 to 11 years was seen.

As expected OA developed during the follow-up period in patients that did not have revision surgery. However the clinical outcome and function scores was improved at a clinical relevant degree. Similar long-term survival findings have been previously reported in younger patient groups [17, 18]. The present study results of clinical relevant outcomes in patients above 65 years may indicate that, with proper patient selection, a mini prosthesis can serve as a long-term treatment modality not only for middle-aged patients [3, 4, 11, 15–18], but also for older patients with significant knee symptoms and impaired function, that do not have severe enough cartilage pathology for UKA/TKA treatment.

As reported, during the full follow-up period, there was a relatively high revision rate of 30% with patients requiring either a UKA or TKA.

An important finding was, that despite a of significant OA progression there were long lasting clinical relevant improvements in pain and function in patients were the implants survived. Also the quality of life EQ-5D score were still high. Also if implants survived the first 5 years, there was evidence for a minimal further risk of revision. This suggests that if symptomatic degenerative cartilage lesions are of limited size rather than an element of generalized OA, then resurfacing implant treatment can provide long-term improvement. The results of 70% not revised to knee arthroplasty after mean 9 years, indicate that resurfacing implants may limit the need for a UKA or TKA in elderly patients (> 65 years old) with symptomatic cartilage lesions or early degenerative knee pathologies [8, 11, 15–18].

Thus far, the present study is the only and largest long-term case series, with 23 femoral resurfacing mini prostheses in elderly knee-patients with clinical and radiographic follow-up up to 11 years, including revision and survival rates.

The strengths of this study included the follow-up duration of up to 11 years and the comprehensive data concerning the revisions, which was a consequence of having a national registry.

A prospective case series such as the present study yields heterogeneous patient material with respect to the cartilage pathology and previous surgery. This study population that might be typical for the patient population suffering significantly from symptomatic cartilage lesions even in elderly patients.

The study was limited by moderate patient numbers and that it was a single-centre case series study with only one operating surgeon, which also was the clinical investigator. This weakens the external validity of the study. There was a low clinical follow-up rate caused by death, revision surgery and high patient-age making them unable to participate in follow-up examination but among those not revised.

## Conclusions

Resurfacing implant treatment for early OA in patients above 65 years can require revision to knee arthroplasty in 30% of patients. But in patients that are not revised long-term improvements in subjective clinical outcome was demonstrated. This suggests that even elderly patients with isolated cartilage lesions or early OA might benefit from the limited invasive resurfacing implant treatment. Only a moderate OA development was found after 10 years and in combination with 70% of patients not needing revision to arthroplasty could indicate that resurfacing implant treatment might delay the need arthroplasty treatment.
